# Bone marrow-derived mononuclear cell therapy can attenuate systemic inflammation in rat heatstroke

**DOI:** 10.1186/s13049-018-0566-2

**Published:** 2018-11-16

**Authors:** Yutaka Umemura, Hiroshi Ogura, Hiroshi Matsuura, Takeshi Ebihara, Kentaro Shimizu, Takeshi Shimazu

**Affiliations:** Department of Traumatology and Acute Critical Medicine, Osaka University Graduate School of Medicine, 2-15 Yamadaoka, Suita, Osaka, 565-0871 Japan

**Keywords:** Heat shock, Inflammation, Multiple organ dysfunction, Stem cells, Transplantation

## Abstract

**Background:**

This study was performed to gain insights into novel therapeutic approaches for acute systemic inflammation in heatstroke. Bone marrow-derived mononuclear cells (BMMNCs) secrete anti-inflammatory proteins and have protective effects against acute inflammation. Recent evidence suggested that transplantation of BMMNCs can reduce the acute tissue injury caused by regional myocardial reperfusion and the lung dysfunction induced by lipopolysaccharides. We evaluated whether BMMNCs attenuate systemic inflammatory response induced by severe heatstroke.

**Material and methods:**

Anesthetized 12-week-old male Wistar rats were subjected to heat stress (41.8 °C for 30 min) with/without transplantation of BMMNCs. Bone marrow cells were harvested from the femur and tibia of other Wistar rats. BMMNCs were separated by density centrifugation, dissolved in phosphate-buffered saline (PBS), and injected intravenously immediately after heat stress (HS-BMMNCs group). The control group was administered an equal volume of PBS, and the sham group underwent the same procedure without heat stress.

**Results:**

Seven-day survival improved significantly in the HS-BMMNCs group versus control group (83.3% vs 41.7%). Transplantation of BMMNCs significantly suppressed serum levels of pro-inflammatory mediators, such as tumor necrosis factor-alpha, interleukin-6 and histone H3 at 3, 6, and 12 h after heat stress. Besides, the elevation of serum syndecan-1, a main component of the vascular endothelial glycocalyx layer, in the BMMNCs group was significantly suppressed compared to that in the control group at 6 and 12 h after heat stress. Histological analysis revealed that edema of the alveolar septum and vascular endothelial injury in the lung were evident in the control group 6 h after heat stress, whereas the morphological alteration was ameliorated in the HS-BMMNCs group. Also, histological analysis using BMMNCs derived from green fluorescent protein transgenic rats showed that the transplanted BMMNCs migrated into lung, kidney, and spleen at 24 h after heat stress but did not engraft to host tissues.

**Conclusion:**

Transplantation of BMMNCs attenuated acute systemic inflammation and vascular endothelial injury, reduced organ dysfunction, and improved survival in a rat heatstroke model. These findings provide a possible therapeutic strategy against critical heatstroke.

## Background

Non-exertional heatstroke is a life-threatening condition caused by prolonged exposure to high temperature and characterized by hyperthermia associated with a systemic inflammatory response leading to multiple organ dysfunction and subsequent death [1–3]. Long-term mortality rates in severe heatstroke patients were reported to be approximately 40–70% [4, 5], and more than 3000 deaths were attributed to heatstroke in the USA from 2006 to 2010 [6]. Although various strategies to control the systemic inflammation induced by heatstroke have been evaluated over the past several decades, the efficacy of therapeutic interventions has not been proved. Thus, a novel therapeutic strategy other than supportive care is required to improve patient outcome.

In the 1950s, bone marrow transplantation was developed as an epoch-making therapeutic method for patients with leukemia [7]. Bone marrow-derived mononuclear cells (BMMNCs) are a heterogeneous group of bone marrow-derived cells consisting of varying proportions of differentially matured B cells, T cells, monocytes, and a smaller proportion of progenitor cells. Recently, the transplantation of BMMNCs has attracted a great deal of attention as a possible therapeutic approach for various clinical targets including the ischemic heart diseases, type 2 diabetes mellitus, and peripheral arterial disease [8–11]. Multiple lines of evidence suggested that BMMNCs had host-protective paracrine properties, and they were reported to reduce organ dysfunction caused by ischemic reperfusion and systemic inflammation in several animal studies [12–15]. Besides, BMMNCs contain a variety of progenitor cells including hematopoietic stem cells, mesenchymal stem cells, and endothelial progenitor cells, which have been reported to support regenerative properties against biological stresses [16, 17].

Systemic inflammation and multiple organ dysfunction play key roles in the pathophysiology of severe heatstroke. In the present experimental study, we transplanted BMMNCs into a rat heatstroke model to evaluate whether and how the transplantation of BMMNCs attenuated biological stresses induced by severe heatstroke. We also pursued the short- and long-term distribution of transplanted BMMNCs in host organs using green fluorescent protein (GFP) transgenic rats.

## Materials and methods

This study was approved by the Animal Care and Use Committee of the Osaka University Graduate School of Medicine, Suita, Japan (Record no: 30–025-000).

### Animals

Specific pathogen-free 12-week-old male Wistar rats weighing 250–300 g were obtained from Nihon SLC (Hamamatsu, Japan). We also used 12-week-old male GFP transgenic rats obtained from Nihon SLC to harvest GFP-positive BMMNCs. Rats were anesthetized by subcutaneous injection of medetomidine (0.375 mg/kg body weight), midazolam (2.0 mg/kg body weight), and butorphanol (2.5 mg/kg body weight). All animal experiments were conducted in accordance with the guidelines of the Animal Care and Use Committee of Osaka University Graduate School of Medicine and were approved by that committee.

### Induction of heat stress

**Fig. 1:**
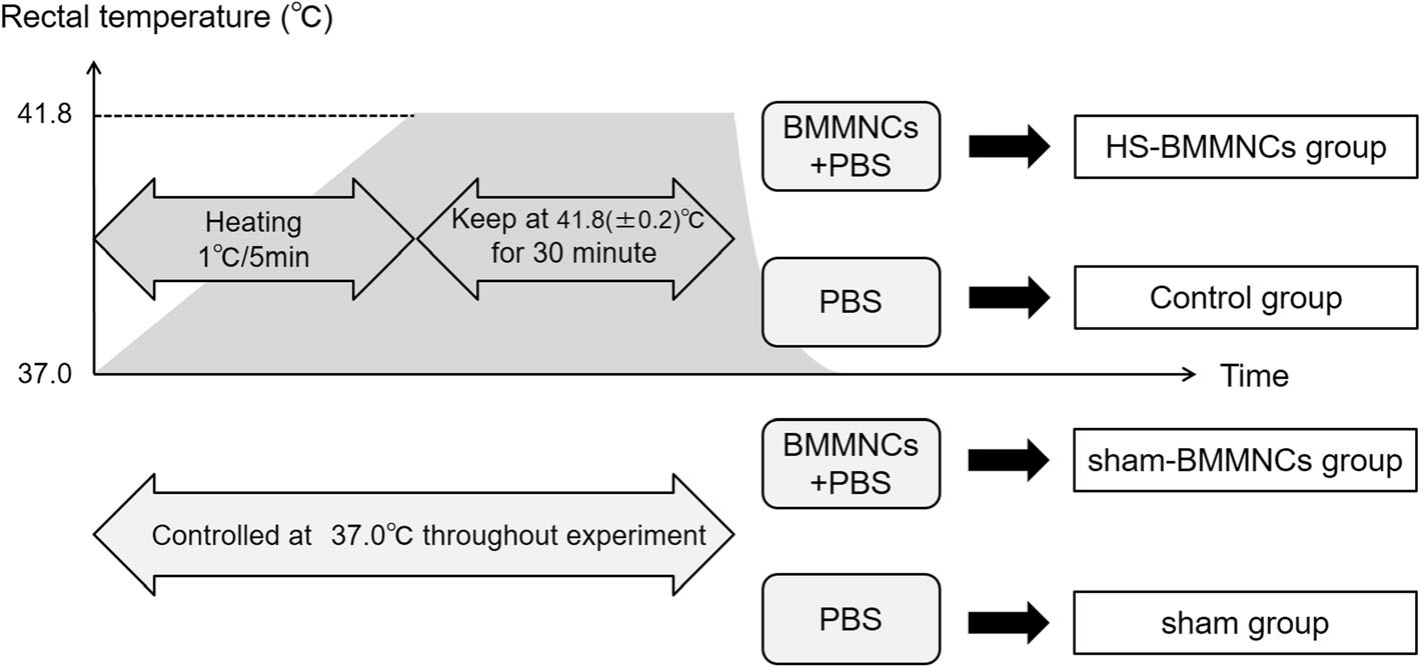
Schema of experimental design of the rat heatstroke model. HS, heatstroke; BMMNCs, bone marrow-derived mononuclear cells; PBS, phosphate-buffered saline

Rat rectal temperatures were monitored continuously throughout the induction of heat stress. Anesthetized rats were subjected to environmental heat stress using a temperature control device with a warm blanket (BWT-100A; Bio Research Center, Nagoya, Japan). The time schedule in this experimental study is shown in Fig 1. The murine model of heatstroke used in this study has been detailed previously [18, 19]. Before induction of heat stress, the rectal temperature of the anesthetized rats was maintained at about 37.0 °C with a heating pad. Next, heat stress was induced by increasing the rectal temperature approximately 1.0 °C every 5 min. From the instant the rectal temperature reached 41.8 °C, the rectal temperature was maintained at 41.8 ± 0.2 °C for 30 min. Then, the heating pad was removed, and the rats were allowed to recover at room temperature (24.0 °C). Finally, saline was injected subcutaneously at a dose of 35 mL/kg to prevent dehydration. The time at which the rats were removed from the temperature control device was defined as 0 h.

### Isolation of BMMNCs

BMMNCs were isolated from the other 12-week-old Wistar rats and GFP transgenic rats. Bone marrows were harvested from femoral and tibial bones. BMMNCs were separated by density centrifugation (400×*g* for 30 min at 20 °C) with a commercially available density solution (Ficoll-Paque TM PREMIUM 1.084; GE Healthcare UK, Ltd., Buckinghamshire, UK) and were re-suspended in PBS. BMMNCs were counted in a Neubauer chamber with Trypan Blue for evaluation of viability.

### Experimental design

Before the induction of heat stress, rats were randomly allocated into the following four groups: i) the Sham group, ii) Sham-BMMNCs group, iii) control (heat stress) group, and iv) heat stress (HS)-BMMNCs group. A cell suspension of 5.0 × 10^6^ viable BMMNCs, which was dissolved in 250 μL of PBS, was injected intravenously into HS-BMMNCs group rats immediately after heat stress. The Sham-BMMNCs group underwent the same procedure without heat stress. The control group and the Sham group were administered equal volumes of PBS. The rats were observed over the 7 days after heat stress to estimate survival rates (*n* = 12 each in the control and HS-BMMNCs groups and *n* = 6 each in the Sham and Sham-BMMNCs groups).

Separate animals were used for blood sampling and histological analysis. Blood samples were collected at 3, 6, and 12 h after heat stress (*n* = 4 in the control group and *n* = 6 in the HS-BMMNCs group at 3 h, *n* = 8 in the control group and n = 6 in the HS-BMMNCs group at 6 h, *n* = 7 in the control group and *n* = 7 in the HS-BMMNCs group at 12 h, and *n* = 5 each in the Sham group and the Sham-BMMNCs group for every time point). The serum was isolated by centrifugation at 3000×*g* for 30 min at 4 °C and frozen at − 80 °C until measurement.

### Measurement of serum levels of mediators and markers

Serum levels of interleukin-1 beta (IL-1β), interleukin-6 (IL-6), tumor necrosis factor alpha (TNF-α), histone-H3, intercellular adhesion molecule (ICAM-1), and syndecan-1 were measured using commercially available enzyme-linked immunosorbent assay kits (IL-1β, IL-6, and ICAM-1: R&D Systems, Minneapolis, MN; TNF-α: abcam, Cambridge, UK; syndecan-1: CUSABIO TECHNOLOGY LLC, Houston, TX; histone H3: LifeSpan BioSciences, Seattle, WA).

### Histological examinations

#### Tissue fixation

Perfusion fixation was carried out to prepare tissue specimens at 6 h after heat stress (*n* = 3 each in the Sham, Control, and HS-BMMNCs groups, respectively). At 6 h after the completion of heat stress, the rats were re-anesthetized and transcardially perfused with PBS followed by 4% paraformaldehyde in 0.1 M phosphate buffer (PB). The tissues were dissected and immersed in the same fixative at 4 °C for 6 h and cryoprotected in a series of sucrose solutions (15, 20, and 25% sucrose in 0.1 M PB) at 4 °C for 3 d. After the specimens were frozen in OCT compound (Sakura Finetechnical Co. Ltd., Osaka, Japan), they were sliced into 7-μm-thick sections by cryostat (CM3050S; Leica Microsystems, Wetzlar, Germany), and the sections were mounted on slides for staining.

#### Hematoxylin-eosin staining

The 7-μm-thick slices of right lung were stained with hematoxylin-eosin and observed under an optical microscope (BZ-9000; Keyence Co., Osaka, Japan).

#### Fluorescent immunostaining

The sections were blocked by 20% Block Ace (Dainippon Sumitomo Pharma Co., Osaka, Japan) in 0.1 M PB with 0.005% saponin (Sigma-Aldrich Co., St. Louis, MO) and incubated with the primary antibody, rabbit monoclonal anti-syndecan-1 antibody (EPR6454; abcam), followed by the secondary antibody, goat anti-rabbit IgG antibody (Goat Anti-Rabbit IgG H&L [HRP]; abcam), overnight at 4 °C. The antibodies were dissolved in 0.1 M PB containing 5% Block Ace and 0.005% saponin. The sections were washed with PBS after each reaction. Finally, they were mounted in 0.1 M PB-glycerin (1:1) solution and observed under a fluorescent microscope (BZ-9000; Keyence Co.). For negative control, nonimmune serum was substituted for the primary antibody.

To investigate the distribution and engrafting of transplanted BMMNCs in the host tissues, we also injected GFP-derived BMMNCs into the heatstroke rats (GFP-BMMNCs group, *n* = 9). Sections of lung, kidney, and spleen were observed under a fluorescent microscope (BZ-9000; Keyence Co.) at 1 day (*n* = 3), 1 week (*n* = 3), and 2 weeks (*n* = 3) after heat stress.

### Statistical analysis

Descriptive statistics are summarized as group means with standard error for continuous variables and frequencies with percentages for categorical variables. Univariate differences between groups were assessed using the Student *t*-test. Survival curves were constructed by the Kaplan-Meier method and compared by log-rank test. All hypotheses were two-sided, and a *p* value of < 0.05 indicated statistical significance. All statistical analyses were conducted using STATA Data Analysis and Statistical Software version 14.0 (StataCorp, College Station, TX).

## Results

### Survival analysis

**Fig. 2:**
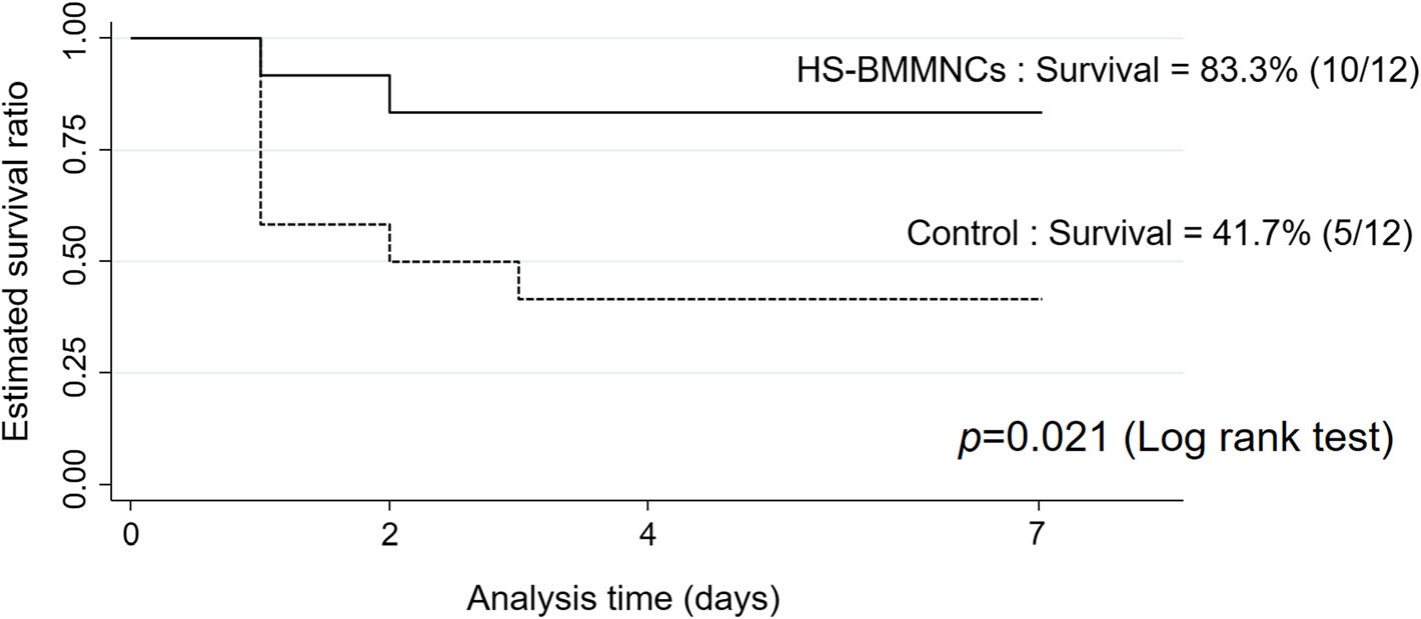
Survival curves by the Kaplan-Meier method over the 7 days after heat stress. The dotted line represents the survival curve of the rats that underwent heat stress without the transplantation of BMMNCs, and the solid line represents the heatstroke rats transplanted with BMMNCs (each group, *n* = 12). Transplantation of BMMNCs significantly improved survival (*p* = 0.021). Sham operation only and sham operation with transplantation of BMMNCs caused no deaths (each group, *n* = 6, data not shown). HS, heatstroke; BMMNCs, bone marrow-derived mononuclear cells

The survival curves derived using the Kaplan-Meier method are shown in Fig 2. All rats in the Sham and Sham-BMMNCs groups survived at 7 days after anesthetization. In the HS-BMMNCs group, the survival rate at 7 days after heat stress was 83.3% (10 of 12 rats) and was significantly higher than that in the control group (41.7%, 5 of 12 rats, *p* = 0.021 by log-rank test).

### Serum levels of inflammatory mediators after heat stress

**Fig. 3:**
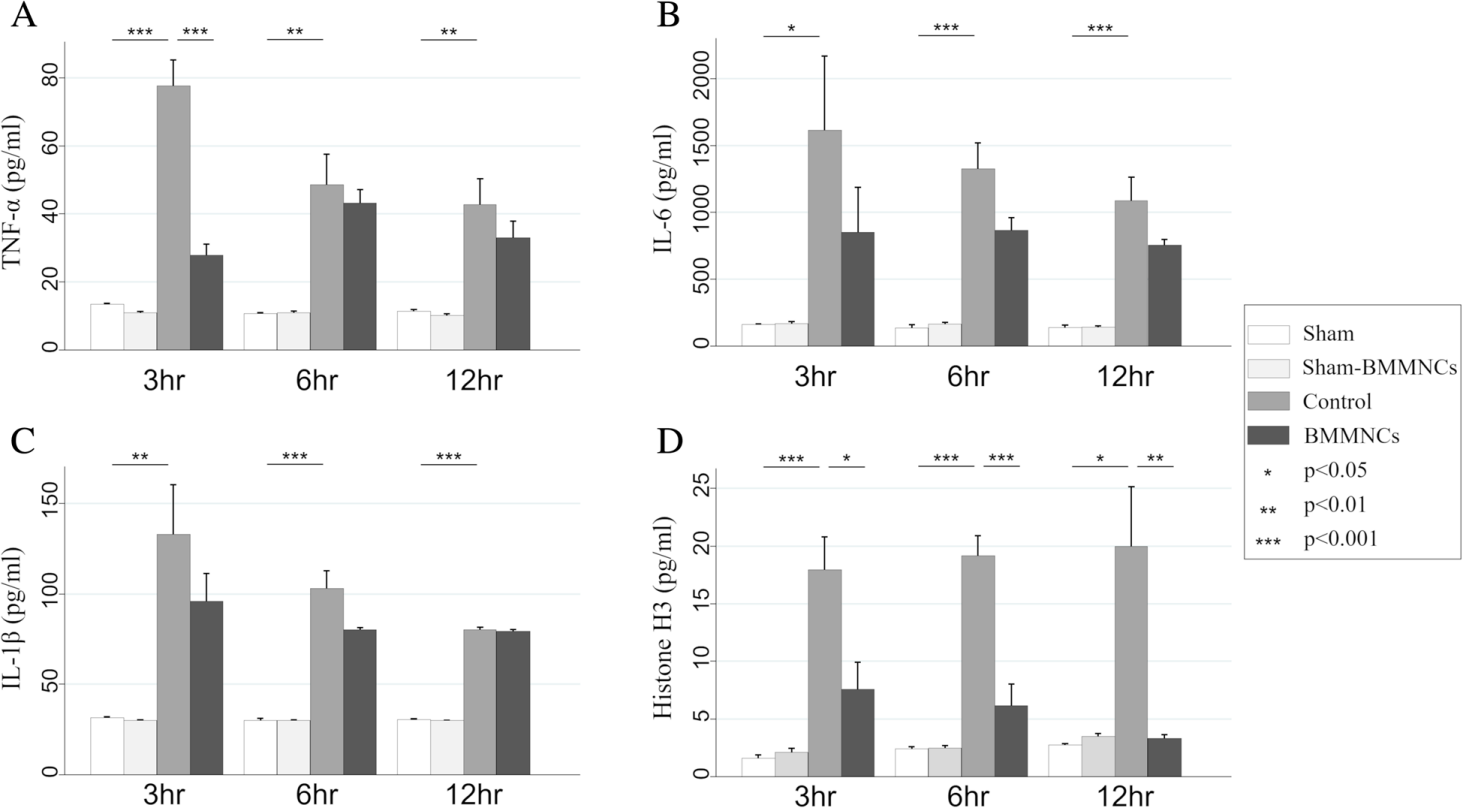
Serial changes in serum levels of TNF-α, IL-6, IL-1β, and histone H3 after heat stress. Serum levels of TNF-α (**a**) IL-6 (**b**) IL-1β (**c**) and histone H3 (**d**) in the sham, sham-BMMNCs, control (heatstroke rats without BMMNCs), and HS-BMMNCs groups at 3, 6, and 12 h after heat stress are shown. All data are expressed as mean with standard error of the mean. TNF, tumor necrosis factor; IL, interleukin; HS, heatstroke; BMMNCs, bone marrow-derived mononuclear cells

We show the serum levels of inflammatory mediators in Fig 3. The serum levels of TNF-α, IL-6, IL-1β, and histone H3 in the control group were significantly higher than those in the Sham group at every time point. The serum level of TNF-α in the HS-BMMNCs group was significantly lower than that in the control group at 3 h after heat stress (*p* < 0.001), whereas there was no significant difference between the two groups at 6 and 12 h. The mean levels of serum IL-6 and IL-1β in the BMMNCs group were lower than those in the control group at every time point, but the differences did not reach statistical significance. The serum levels of histone H3 in the control group gradually increased after heat stress. On the contrary, the serum levels of histone H3 in the BMMNCs group gradually decreased and were significantly lower at every time point (*p* = 0.019, p < 0.001, and *p* = 0.007, respectively).

### Serum markers of endothelial injury after heat stress

**Fig. 4:**
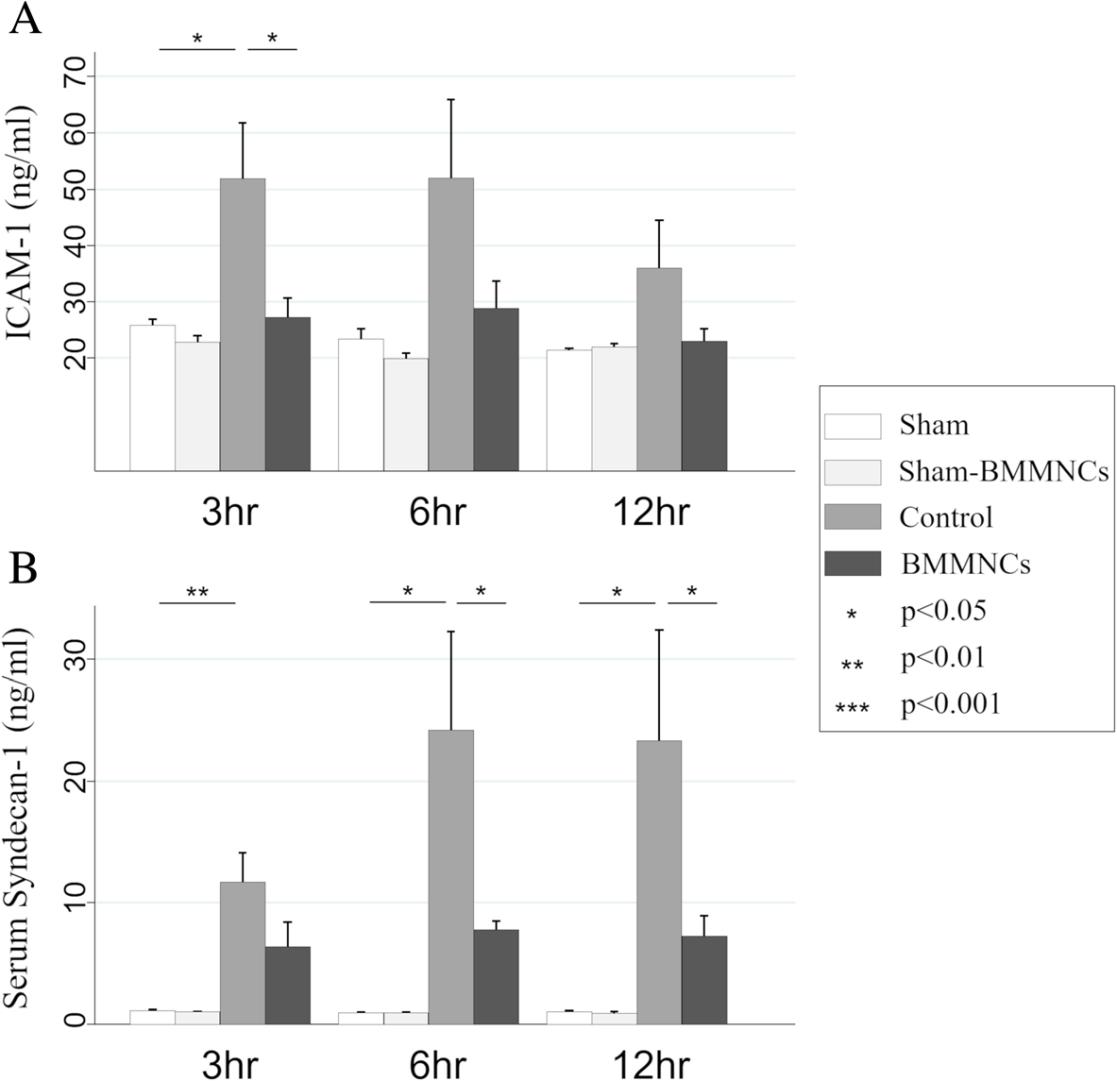
Serial changes in serum levels of ICAM-1 and syndecan-1 after heat stress. Serum levels of ICAM-1 (**a**) and syndecan-1 (**b**) in the sham, sham-BMMNCs, control (heatstroke rats without BMMNCs), and HS-BMMNCs groups at 3, 6, and 12 h after heat stress are shown. All data are expressed as mean with standard error of the mean. ICAM, intercellular adhesion molecule; HS, heatstroke; BMMNCs, bone marrow-derived mononuclear cells

The serum levels of ICAM-1 and syndecan-1 are shown in Fig 4. Although the mean level of serum ICAM-1 in the BMMNCs group was lower than that in control group at every time point, a statistically significant difference was observed only at 3 h after heat stress. The serum level of syndecan-1 in the BMMNCs group was significantly lower than that in the control group at 6 and 12 h after heat stress (*p* = 0.033 and *p* = 0.048, respectively).

### Histological examinations

**Fig. 5:**
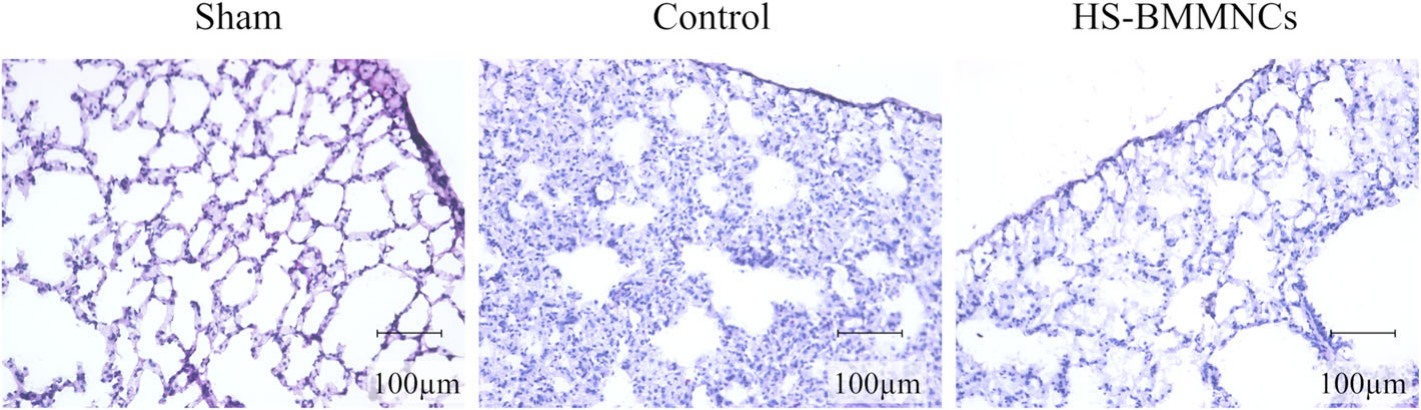
Hematoxylin-eosin-stained sections of the lung in the sham, control, and HS-BMMNCs groups at 6 h after heat stress. Original magnification, 200×. HS, heatstroke; BMMNCs, bone marrow-derived mononuclear cells

Hematoxylin-eosin-stained sections of the lung at 6 h after heat stress are shown in Fig 5. Inflammatory cell infiltration with edema in the alveolar septum was evident in the control group compared to the Sham group, but this morphological alteration was ameliorated in the HS-BMMNCs group.

**Fig. 6:**
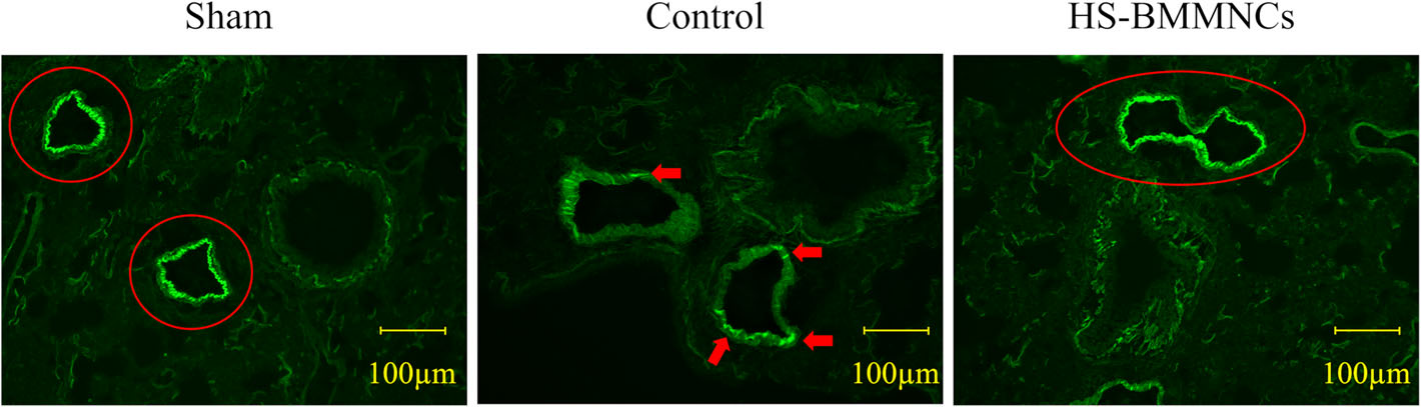
Immunohistochemical expression of syndecan-1 on the vascular surface in the lung at 6 h after heat stress. Arrows or circle indicate the expression of syndecan-1 in the vascular endothelial glycocalyx layer. Original magnification, 200×. HS, heatstroke; BMMNCs, bone marrow-derived mononuclear cells

Immunofluorescence images of lung incubated with polyclonal antibody against syndecan-1, a main component of the vascular endothelial glycocalyx layer, at 6 h after heat stress are shown in Fig 6. The expression of syndecan-1 on the pulmonary vascular surface was dramatically reduced in the Control group compared to that in the Sham group. In contrast, we observed little alteration in the expression of syndecan-1 in the HS-BMMNCs group compared to that in the Sham group.

**Fig. 7:**
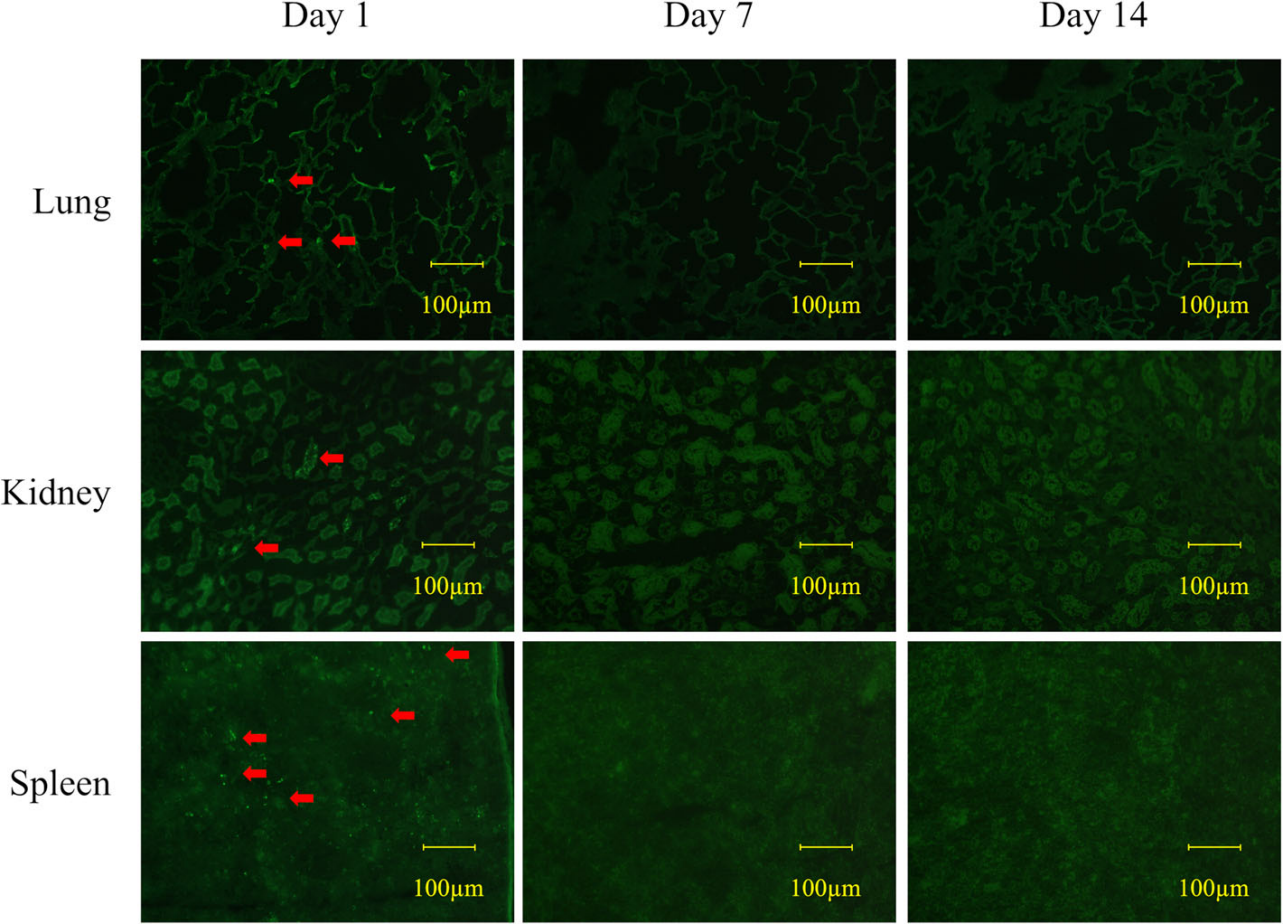
Confocal microscopy of the lung, kidney, and spleen at 1 day, 1 week, and 2 weeks after transplantation of BMMNCs from GFP transgenic rats. Arrows indicate the GFP-positive cells. Original magnification, 200×. GFP, green fluorescent protein; BMMNCs, bone marrow-derived mononuclear cells

At 1 day after heat stress with the transplantation of BMMNCs derived from GFP transgenic rats, we observed small proportions of GFP-positive cells in lung, kidney, and spleen of the GFP-BMMNCs group. However, we observed no GFP-positive cells in any sections of GFP-BMMNCs at either 1 week or 2 weeks after heat stress (Fig 7).

## Discussion

In heatstroke, the combination of thermoregulatory failure, systemic inflammation, circulatory shock, and coagulation disorders can cause multiple organ dysfunction and death. The total number of deaths from heatstroke continues to rise despite advances in supportive care such as fluid resuscitation and whole-body cooling methods [3]. Therefore, a substantial therapeutic strategy against systemic inflammation needs to be established. The present study showed for the first time, to our knowledge, that the transplantation of BMMNCs attenuated systemic inflammatory response, reduced organ dysfunction, and resulted in an increased survival rate in a rat heatstroke model.

Non-exertional severe heatstroke due to passive heat exposure promotes the production of a variety of pro-inflammatory mediators, such as IL-1β, TNF-α, IL-6, and high-mobility group box 1 (HMGB1), which cause an excess activation of leukocytes, endothelial injury, and coagulation disorders that lead to multiple organ dysfunction [20–22]. Multiple lines of evidence suggested that the paracrine property of BMMNCs reduces pro-inflammatory cytokines and contributes to host-protective effects against systemic inflammation [15, 23]. Lovell et al. reported that the transplantation of BMMNCs to a myocardial ischemia and reperfusion model enhanced the expression of the pro-survival phosphoinositide 3-kinase (PI3K)/Akt signaling pathway and reduced infarct size and cardiac dysfunction. AKT is an important PI3K signaling effector that regulates cellular metabolism, inflammatory responses, and cell apoptosis [24]. When phosphorylated by its upstream regulator, AKT phosphorylates were reported to reduce the activation of GSK-3B, resulting in the reduction of systemic inflammation via inhibition of NF-kB [25, 26]. In the present study, decreased levels of pro-inflammatory mediators such as TNF-α and IL-6 might be attributable to these types of anti-inflammatory pathways promoted by the transplantation of BMMNCs.

We also showed that the elevations of histone H3 observed in the heatstroke rats were significantly suppressed by transplantation of BMMNCs, along with the other pro-inflammatory mediators. Histone H3 leaks out from the injured cell and causes endothelial cytotoxicity and organ failure as damage-associated molecular patterns. Besides, histone H3 is known from previous studies [27] to be a main component of neutrophil extracellular traps (NETs) released from activated neutrophils. Therefore, our findings suggested that activation of neutrophils and release of NETs were predominantly accelerated in heatstroke and might be suppressed by the transplantation of BMMNCs.

Vascular endothelium is composed of the endothelial cell monolayer and vascular glycocalyx layer and plays a critical role in vascular permeability, blood coagulation, and host immune responses in systemic inflammation. Extreme heat stress was reported to directly induce endothelial injury [28]. In the present study, we showed for the first time that the transplantation of BMMNCs significantly suppressed the elevation of serum syndecan-1, which is the main component of the inner layer of glycocalyx and is reported to be a major target of circulating histone [29]. Therefore, our findings suggested that the transplantation of BMMNCs could attenuate the injury of the vascular glycocalyx layer, partly by suppressing the release of histone and other pro-inflammatory mediators during systemic inflammation.

BMMNCs have been expected to exert anti-inflammatory and regenerative properties in various organs including lung, kidney, and heart. However, there is insufficient evidence on the distribution of BMMNCs and whether BMMNCs engraft to host tissues in acute systemic inflammation. Ornellas et al. reported that GFP-positive cells were detected in the lung and kidney of CLP (cecal ligation and puncture) mice treated with BMMNCs derived from GFP transgenic mice at day 1, but they were no longer detected at day 7 [30]. Similarly, we detected GFP-positive cells in the lung, kidney, and spleen of the GFP-BMMNCs group at 1 day after heat stress, but they were no longer detectable at 1 week and 2 weeks after heat stress. These findings clearly suggested that the transplanted BMMNCs could migrate to various types of organs and played a critical role in anti-inflammatory properties, but most of them did not engraft to host tissues.

In the present study, we evaluated a possible therapeutic approach to heatstroke, but the evidence was based on an experimental design using an animal model. There are still several significant problems awaiting resolution before BMMNC therapy can be applied in the clinical setting. First, even though the rat heatstroke model we chose is already established, it does not fully mimic human heatstroke. Besides, human heatstroke is a heterogeneous syndrome, partly due to the widely ranging exposure times to heat stresses. Second, this therapy requires healthy donors in the clinical setting because autologous bone marrow in heatstroke patients may be altered by the exposure to heat and acute inflammation. Transplantation of autologous BMMNCs has been clinically evaluated for several diseases, such as traumatic brain injury, spinal cord injury, and ischemic cerebral infarction [31–33], but allogeneic transplantation of BMMNCs has not been conducted clinically. Recently, several facilities established a mesenchymal stem cell bank and are banking these cells for future use in regenerative medicine applications [34]. Furthermore, a number of studies have reported methods of generating mesenchymal stem cells derived from induced pluripotent stem cells [35]. We thus believe that the combination of these epoch-making techniques may play a key role in the future application of BMMNC therapy clinically. BMMNCs have so far been reported to regulate acute inflammation and reduce organ dysfunction in various critical diseases such as sepsis, hemorrhagic shock, and acute respiratory distress syndrome [26, 30, 36]. Therefore, the clinical application of BMMNC therapy could lead to a paradigm shift in the approach to these acute inflammatory diseases.

### Limitations

Several limitations of this study must be mentioned. First, the time schedule of BMMNCs transplantation in this experimental study might differ from the actual clinical situations of heatstroke, in which therapeutic interventions sometimes cannot be conducted immediately after heat stress is suffered. Second, because we used unfractionated BMMNCs, we did not characterize which type of cells among the overall BMMNCs was most responsible for the beneficial effects against heat stress. Therefore, further investigation that includes the effects of delayed transplantation of BMMNCs or the different effects on heatstroke caused by the fractions of BMMNCs is required.

## Conclusion

Transplantation of BMMNCs attenuated acute systemic inflammation, reduced organ dysfunction, and dramatically improved survival in a rat heatstroke model. These findings provide a possible therapeutic strategy against critical heatstroke.

## Abbreviations

BMMNCs: Bone marrow-derived mononuclear cells
GFP: Green fluorescent protein
HMGB1: High-mobility group box 1
HS: Heat stress
ICAM-1: Intercellular adhesion molecule 1
IL-1β: Interleukin-1 beta
IL-6: Interleukin-6
NETs: Neutrophil extracellular traps
PB: Phosphate buffer
PBS: Phosphate-buffered saline
PI3K: Phosphoinositide 3-kinase
TNF-α: Tumor necrosis factor alpha
